# Subacute Thyroiditis Without Neck Pain: An Unusual Cause of Persistent Fever in the Elderly Patient

**DOI:** 10.1002/ccr3.72259

**Published:** 2026-03-10

**Authors:** Abdullah Shahid, Aasim Ali, Javed Iqbal, Muhammad Asad Shabbir, Usama Saleem, Muhammad Shahzeb, Danish Yousuf, Gohar Mushtaq, Armaghana Abdullah, Mukesh Kumar Sharma

**Affiliations:** ^1^ Medicine Department Allied Hospital Faisalabad Pakistan; ^2^ Neurology Department Allied Hospital Faisalabad Pakistan; ^3^ Dhankuta District Hospital Dhankuta Nepal

**Keywords:** de Quervain's thyroiditis, elderly patient, fever of unknown origin, subacute thyroiditis, thyroid function abnormalities

## Abstract

Subacute thyroiditis can present as fever of unknown origin, particularly in elderly patients. Absence of neck pain or thyroid tenderness does not exclude the diagnosis. Thyroid function tests and inflammatory markers are key to diagnosis. Corticosteroids lead to rapid symptomatic improvement in moderate‐to‐severe cases. A transient hypothyroid phase is common and often resolves without long‐term thyroxine therapy.

## 1 Introduction

Subacute (de Quervain's) thyroiditis is an inflammatory disorder of the thyroid gland most often attributed to a post‐viral or viral inflammatory response, typically presenting with fever, anterior neck pain, tenderness, and elevated inflammatory markers [1]. While the classical presentation includes painful thyroid swelling and systemic symptoms including hyperthyroidism, there are increasing reports of atypical forms in which fever or constitutional symptoms predominate and classic neck pain or thyroid tenderness are absent [2]. Fever of unknown origin (FUO) has been described as a rare but important presentation of subacute thyroiditis, particularly in elderly patients, and recognizing this atypical manifestation is crucial to avoid unnecessary extensive investigations and inappropriate antimicrobial therapy [3]. Early consideration of thyroid pathology in the differential diagnosis of prolonged fever and raised inflammatory markers can facilitate timely diagnosis and appropriate anti‐inflammatory treatment.

## 2 Case Presentation

An 80‐year‐old man presented with a 1‐week history of persistent high‐grade fever, documented between 101°F–102°F, which was continuous in nature. The fever was associated with mild discomfort on swallowing (odynophagia). There was no history of cough, sputum production, shortness of breath, rigors, chills, dysuria, urinary frequency, abdominal pain, diarrhea, vomiting, weight loss, night sweats, or recent travel. He did not report neck pain, thyroid tenderness, palpitations, tremors, heat intolerance, or excessive sweating. There was no preceding history suggestive of an upper respiratory tract infection.

On physical examination, the patient was febrile but hemodynamically stable. Oropharyngeal examination revealed mild pharyngeal erythema without tonsillar exudates. There was no cervical lymphadenopathy. Examination of the neck showed no visible thyroid enlargement, and the thyroid gland was non‐tender on palpation. Cardiovascular, respiratory, and abdominal examinations were unremarkable, and no focal neurological deficits were identified.

Initial laboratory investigations showed a white blood cell count of 7000/μL with neutrophilic predominance (65%), markedly elevated inflammatory markers with a C‐reactive protein (CRP) level of 65 mg/L and an erythrocyte sedimentation rate (ESR) of 115 mm/h. Liver and renal function tests were within normal limits. Chest radiograph and urinalysis did not reveal any abnormalities. Blood and urine cultures remained sterile. In view of persistent fever, the patient was empirically started on a third‐generation cephalosporin, which was later escalated to meropenem; however, there was no clinical improvement.

On reevaluation, the patient continued to have high‐grade fever (101°F–102°F) along with persistent mild odynophagia. Repeat laboratory testing showed a white blood cell count of 8000/μL, with further elevation of inflammatory markers (CRP 112 mg/L; ESR 115 mm/h). Thyroid function tests demonstrated a suppressed thyroid‐stimulating hormone (TSH) level of 0.01 μIU/mL, while serum triiodothyronine (T3) and thyroxine (T4) levels were at the upper limit of normal. High‐resolution CT of the chest and neck did not identify any infective or inflammatory focus. Ultrasonography of the thyroid revealed a gland of normal size with heterogeneous echotexture, without focal nodules, collections or abscess formation (All Investigations shown in Table 1 below).

**TABLE 1:** Investigations carried during the course of illness.

Test	Results	Reference range
CRP	61 mg/L	< 5 mg/L
WBCs	6510/mm^3^	/mm^3^
RBCs	4.18 million/mm^3^	4.5–5.5 million/mm^3^
Platelet	202,000/mm^3^	150,000–350,000/mm^3^
X‐ray chest P.A view	Normal	
Serum creatinine	0.8 mg/dL	0.72–1.25 mg/dL
SGOT (AST), SGPT (ALT)	31, 32 U/L	< 50 U/L
Total bilirubin	0.05 mg/dL	< 1.2 mg/dL
HIV, HBV, and HCV serology	Negative	
Blood and urine culture	No bacterial growth isolated	
USG of thyroid gland	Thyroid gland is normal in size but heterogeneous echotexture	
Thyroid function tests (on Day 14 of fever)	T3: 121 ng/dL T4: 10.3 μ/dL TSH: 0.01 μIU/L	80–200 ng/dL 5.1–14.1 μg/dL 0.5–8.9 μIU/L
Thyroid function tests (1 month later)	T3: 67 ng/dL T4: 4.07 μ/dL TSH: 8.65 μIU/L	80–200 ng/dL 4.2–13.0 μg/dL 0.5–8.9 μIU/L
Thyroid peroxidase (TPO) antibodies	Negative	
Thyroglobulin (Tg) antibodies	Negative	
CRP (After 1 month of treatment)	11.6 mg/L	< 5 mg/L
Echocardiography	Normal study	
USG abdomen and KUB	Unremarkable	

## 3 Differential Diagnosis

The differential diagnoses included occult bacterial infection, viral pharyngitis, giant cell arteritis, thyroiditis, and drug‐induced fever. The absence of localizing signs, sterile cultures, lack of response to antibiotics, and characteristic thyroid function abnormalities supported the diagnosis of subacute thyroiditis. Silent thyrioditis was also ruled out as thyroid peroxidase (TPO) antibodies and thyroglobulin (Tg) antibodies were negative.

## 4 Treatment

The patient was initially started on nonsteroidal anti‐inflammatory drugs. Due to persistent symptoms, oral prednisolone was commenced at a dose of 20 mg/day.

## 5 Outcome and Follow‐Up

The patient showed marked clinical improvement within 48 h of initiating corticosteroid therapy, with complete resolution of fever and odynophagia. Prednisolone was gradually tapered over 3 weeks. At follow‐up after 3–4 weeks, the patient was asymptomatic and inflammatory markers had normalized.

Repeat thyroid function testing demonstrated a transient hypothyroid phase, with TSH of 8.75 μIU/mL and low–normal T3 and T4 levels. As the patient remained clinically euthyroid, levothyroxine replacement therapy was not initiated; moreover, this subsequent hypothyroidism is expected to be transient, supporting the decision not to initiate levothyroxine therapy. Serial thyroid function tests were planned every 6–8 weeks.

Each time total T3 and total T4 were measured through chemiluminescence immunoassay (CLIA) method but in different units.

## 6 Discussion

Subacute (de Quervain's) thyroiditis is traditionally described as an inflammatory disorder of the thyroid gland that presents with anterior neck pain, tenderness, and systemic symptoms including fever and thyrotoxic features [4]. Although it most commonly affects middle‐aged adults—particularly women in their fourth and fifth decades—cases have been reported across a wide age spectrum, including in elderly patients, highlighting that age should not exclude the diagnosis [2]. Indeed, the index patient in this report was 80 years old, which aligns with other documented cases where advanced age contributed to diagnostic difficulty due to atypical symptomatology [5].

A hallmark feature of subacute thyroiditis has historically been pain in the anterior neck region due to thyroid capsule inflammation; however, recent evidence indicates that a subset of patients present without this classical symptom. Reports describe “painless” subacute thyroiditis and cases lacking thyroid tenderness on examination, particularly when fever or constitutional symptoms predominate [6]. Absence of neck pain can frequently mislead clinicians, especially when the presentation mimics FUO—a diagnostic category that encompasses persistent fever without localizing signs after an extensive workup. In such scenarios, classical infectious, rheumatologic, and neoplastic causes are typically investigated first, often delaying consideration of endocrine causes like subacute thyroiditis [3].

The elderly population may be particularly prone to atypical presentations. Age‐related blunting of pain perceptions, absence of robust inflammatory manifestations, and coexisting comorbidities can mask classic symptoms, such as neck discomfort, leading to prolonged fever as the sole or predominant complaint [7]. In published cases of elderly individuals with subacute thyroiditis presenting as FUO, the lack of neck pain was a consistent feature, and diagnosis was often only reached after persistent investigation and exclusion of more common causes of fever [8]. This emphasizes the importance of including thyroid function tests and inflammatory marker evaluation early in the diagnostic pathway for FUO, especially in older patients with unexplained fever and elevated ESR or CRP. The initial biochemical findings in subacute thyroiditis reflect a destructive thyrotoxicosis rather than true hyperthyroidism.

Furthermore, contemporary literature suggests that atypical presentations, including painless cases and those with FUO, may be underrecognized. A systematic review and bibliometric analysis highlight emerging patterns of subacute thyroiditis presentations that deviate from the traditional pain‐dominant picture, potentially influenced by factors such as analgesic use, concurrent infections, or age‐related changes in immune response [4]. These atypical forms underscore the need for clinicians to maintain a high index of suspicion in patients with elevated inflammatory markers and suppressed TSH, even in the absence of neck pain or classical thyrotoxic signs.

Overall, this case illustrates that subacute thyroiditis should be considered in the differential diagnosis of FUO, particularly in elderly patients. Early recognition and appropriate steroid therapy can lead to rapid symptomatic improvement and prevent unnecessary investigations and prolonged empirical antimicrobial use.

## Author Contributions

**Abdullah Shahid:** writing – original draft. **Aasim Ali:** writing – original draft. **Javed Iqbal:** data curation, supervision. **Muhammad Asad Shabbir:** data curation. **Usama Saleem:** supervision, writing – review and editing. **Muhammad Shahzeb:** software, validation. **Danish Yousuf:** supervision, writing – review and editing. **Gohar Mushtaq:** data curation, validation. **Armaghana Abdullah:** supervision, writing – review and editing. **Mukesh Kumar Sharma:** software.

## Funding

The authors have nothing to report.

## Consent

A written and informed consent was obtained from the patient to publish her clinical health related information.

## Conflicts of Interest

The authors declare no conflicts of interest.

## Data Availability

Data sharing not applicable to this article as no datasets were generated or analysed during the current study.
